# Synergistic role of 5-azacytidine and ascorbic acid in directing cardiosphere derived cells to cardiomyocytes *in vitro* by downregulating Wnt signaling pathway via phosphorylation of β-catenin

**DOI:** 10.1371/journal.pone.0188805

**Published:** 2017-11-30

**Authors:** Reddy Sailaja Mundre, Pavani Koka, Prakash Dhanaraj, Nitin Khatri, Sanjana Vig, Yamini Chandramohan, Anuradha Dhanasekaran

**Affiliations:** Centre for Biotechnology, Anna University, Chennai, Tamil Nadu, India; University of Kansas Medical Center, UNITED STATES

## Abstract

**Background:**

Cardiosphere derived cells (CDCs) represent a valuable source in stem cell based therapy for cardiovascular diseases, yet poor differentiation rate hinders the transplantation efficiency. The aim of this study is to check the ability of 5-Azacytidine (Aza) alone and in combination with ascorbic acid (Aza+AA) in delineating CDCs to cardiomyogenesis and the underlying Wnt signaling mechanism in induced differentiation.

**Methods:**

CDCs were treated with Aza and Aza+AA for a period of 14 days to examine the expression of cardiac specific markers and Wnt downstream regulators by immunofluorescence, real time PCR and western blot.

**Results:**

Results revealed that Aza+AA induced efficient commitment of CDCs to cardiomyogenic lineage. Immunofluorescence analysis showed significant augment for Nkx 2.5, GATA 4 and α-Sarcomeric actinin markers in Aza+AA group than control group (*p* = 0.0118, *p* = 0.009 and *p* = 0.0091, respectively). Relative upregulation of cardiac markers, Nkx 2.5 (*p* = 0.0156), GATA 4 (*p =* 0.0087) and down regulation of Wnt markers, β-catenin (*p* = 0.0107) and Cyclin D1 (*p =* 0. 0116) in Aza+AA group was revealed by RNA expression analysis. Moreover, the Aza+AA induced prominent expression of GATA 4, α-Sarcomeric actinin and phospho β-catenin while non phospho β-catenin and Cyclin D1 expression was significantly suppressed as displayed in protein expression analysis. Generation of spontaneous beating in Aza+AA treated CDCs further reinforced that Aza+AA accelerates the cardiomyogenic potential of CDCs.

**Conclusion:**

Combined treatment of Aza along with AA implicit in inducing cardiomyogenic potential of CDCs and is associated with down regulating Wnt signaling pathway. Altogether, CDCs represent a valuable tool for the treatment of cardiovascular disorders.

## Introduction

Myocardial infarction with irreversible loss of functional cardiomyocytes remains the foremost cause for cardiovascular related mortality worldwide. World Health Organization (WHO) anticipates around 24 million deaths due to cardiovascular diseases (CVDs) in the coming decade [1]. Currently, regeneration of damaged cardiac tissue with functional cardiomyocytes via stem cell therapy represents an effective approach in CVDs treatment. The discovery of endogenous cardiac progenitor cells (CPCs) in the heart by Beltrami et al., in 2003 has revolutionized cardiac regenerative field [2]. Since then, various CPC sub populations were identified and their cardiomyogenic potential was testified by many groups [3–7]. Among these, Cardiosphere derived cells (CDCs) are therapeutically advantageous because of the properties like immuno compatibility, superior paracrine activity with multipotent nature to give rise to cardiac lineage, better engraftment and synchronization with the surrounding myocardium upon transplantation [8–12].

Phase 1 clinical trial, CADUCEUS (CArdiosphere-Derived aUtologous stem CElls to reverse ventricUlar dySfunction) using CDCs demonstrated reduced infarct size and increased viable myocardium. Nonetheless, there was no significant increase in left ventricular ejection fraction (LVEF) and no evidence that CDCs were differentiated to cardiomyocytes [13]. Yet, the hurdles like low survival rate, poor engraftment and inadequate differentiation of transplanted stem cells are to be addressed for a successful clinical outcome. Stimulation and pre differentiation of stem cells *in vitro* is the viable option for effective myocardial regeneration when transplanted *in vivo*. Moreover, further investigation into the molecular mechanism involved in cardiomyogenesis may strengthen the efficiency of cell based therapeutics.

Small molecules like 5-azacytidine (Aza), oxytocin, dexamethasone and retinoic acid can induce differentiation of stem cells into cardiomyocytes [14]. Among these, Aza is known to stimulate cardiomyogenesis through epigenetic reprogramming in CPCs and other stem cells [15–17]. Ascorbic acid (AA), a well-known cofactor in biological processes can influence proliferation and differentiation of stem cells [18–20]. Wnt signalling has been implicated as an essential regulator of cardiac morphogenesis, progenitor cell proliferation and differentiation [21]. Wnt shows biphasic character during embryo development, activating cardiac gene expression at the early stage of mesoderm formation and suppressing cardiac induction at later stages. [22, 23]. Reports also suggest augmented levels of Wnt signaling during cardiac injury and rejuvenation process [24]. Understanding the regulation of Wnt signaling by small molecules involved in delineating CDCs towards cardiomyogenesis is crucial for successful outcome in myocardial therapeutics.

Taking into account i) CDCs in-built competence to give rise to cardiac lineage ii) Aza, being a known inducer of mesodermal lineage, cardiomyogenesis in particular and iii) ability of AA in promoting cardiomyogenic lineage, the present study was designed to investigate the effects of Aza alone and in combination with AA in mediating cardiomyogenic differentiation of CDCs and modulation on Wnt signaling.

## Materials and methods

### Materials

The materials required to perform the study were purchased from Abcam, USA (Rabbit monoclonal GATA 4 antibody), Cell signaling technology, USA (Rabbit monoclonal antibodies - α-Sarcomeric actinin, β-catenin and Cyclin D1), Invitrogen, USA (IMDM, DMEM/F12, FBS, Trypsin-EDTA, PBS, Bovine Serum Albumin, B27 supplement, L-Glutamine, Recombinant human EGF, Recombinant human basic bFGF, Recombinant human cardiotrophin-1), Sigma-Aldrich, USA (Fibronectin, poly-D-lysine, Thrombin, Paraformaldehyde, 5-Azacytidine, ascorbic acid, 2-mercaptoethanol, Anti-rat IgG-FITC (Fluorescein isothiocyanate), Mouse Monoclonal Anti-α-Sarcomeric actinin antibody, DAPI, TRIzol solution), Thermo Fisher Scientific, USA (Revert aid first strand cDNA synthesis kit), KAPA Biosystems, USA (KAPA SYBR® FAST One-Step qRT-PCR Master Mix (2X) Kit), Santa Cruz Biotechnology, USA (Bradford reagent, Rabbit polyclonal c-Kit antibody, Rabbit polyclonal Nkx 2.5 antibody, mouse monoclonal GATA 4 antibody and goat anti-rabbit PE (phycoerythrin)-conjugated secondary antibody, goat antirabbit HRP conjugated secondary antibody), Bio legend (CD 105 antibody), Cayman, USA (LDH cytotoxicity assay kit), GE healthcare life sciences, India (Amersham ECL Western Blotting Detection Reagent, PVDF membrane). All working solutions were prepared using tissue culture grade water under sterile conditions.

### Ethics approval

All the procedures involved in this study were reviewed and approved by Anna University Institutional Animal Ethics Committee (Ref No: CBT/AU/IAEC/02/2016) in strict accordance with the guidelines of Committee for the Purpose of Control and Supervision on Experiments on Animals (CPCSEA), India.

### Derivation of CDCs from rodent heart by explant culture

CDCs were expanded from the whole hearts of male Wistar rats of 1-month old as described [7] with minor modifications. In brief, the hearts were excised from anesthetized rats and perfused with Ca^2+^-Mg^2+^ free phosphate buffered saline (PBS) several times and dissected into 1–2 mm^3^ fragments, washed and partially digested thrice with 0.05% trypsin [25]. These tissue fragments were cultured in fibronectin-coated 6 well plates in Complete Explant Medium (CEM) (Iscove’s Modified Dulbecco’s Medium [IMDM] supplemented with 15% Fetal Bovine Serum (FBS), 100 U/mL penicillin-streptomycin, 2 mmol/L L-glutamine, and 0.1 mmol/L 2-mercaptoethanol). Upon confluency, cardiac explant outgrowth cells (CEOCs) were harvested by treating with 0.05% trypsin and seeded at a density of 4x10^4^ cells/well in poly-D-lysine-coated 24 well plates in Cardiosphere Growth Medium (CGM) (35% IMDM/65% DMEM–Ham F-12 mixed with 2% B27, 0.1 mmol/L 2-mercaptoethanol, 10 ng/mL epidermal growth factor (EGF), 20 ng/mL basic fibroblast growth factor (bFGF), 40 nmol/L cardiotrophin-1, 40 nmol/L thrombin, 2 mmol/L L-Glutamine and 100 U/mL penicillin-streptomycin). Cells that adhered to the plate were discarded, while cardiospheres formed in suspension were collected and replated onto fibronectin-coated T-25 flasks to generate a monolayer of CDCs. CDCs from passages 2–3 were utilized for all the experiments.

### Characterization of CEOCs and CDCs by immunofluorescence analysis

Immunofluorescence analysis of CEOCs and CDCs were carried out according to the established protocol of our lab [26, 27]. Cardiac explants and CDCs were cultured directly on fibronectin coated glass cover slips for immunofluorescence analysis. CEOCS obtained from explants were analyzed for c-kit marker, specific to CPCs and CDCs were analyzed for surface markers, c-kit and CD 105 (specific to CDCs). Cells were fixed with 4% paraformaldehyde for 15 minutes and blocked in blocking buffer (1% BSA in PBS) for 30 minutes at room temperature. Cells were then incubated with primary antibodies, c-kit and CD 105 (1:200 dilution in blocking buffer) overnight at 4°C. Following three PBS washes, cells were incubated with respective fluorescence conjugated secondary antibodies (1:1000) for 1 hour at room temperature. DAPI was used to counterstain nuclei and the cells were visualized using confocal microscope (Carl Zeiss, Zen 2010) with appropriate filters.

### Optimization of Aza and AA concentration for differentiation induction of CDCs

#### LDH cytotoxicity assay

To optimize the Aza concentration, CDCs were seeded at a density of 5x10^3^ cells/well in a 96 well plate and were treated with Aza at five different concentrations of 1, 5, 10, 20, 50 μM for two days. Lactate dehydrogenase (LDH) release from the cells was measured using LDH cytotoxicity assay kit (Cayman chemicals, USA) on day 1 and day 2 respectively. The percentage of cell death was calculated according to the formula provided in the assay kit.

#### Effect of different concentrations of Aza on cell proliferation of CDCs by Alamar blue assay

CDCs were plated onto 96 well culture plates at the density of 5×10^3^ cells/well in 100μl IMDM supplemented with 10% FBS. Following 24 hours of culture, the cells were incubated with three different concentrations, 1, 5 and 10μM of Aza and cell proliferation was measured at the intervals of 1, 2 and 3 days respectively. Percentage of proliferation was calculated using the formula provided in the assay kit.

#### Determination of AA concentration for differentiation induction of CDCs

CDCs were seeded in 12 well plates at the density of 5x10^3^ cells/well in IMDM containing 10% FBS. After 24 hours, CDCs were treated with 10, 50, 100, 250, 500 μM concentrations of AA. The effect of AA on proliferation of CDCs was measured by counting cells manually using trypan blue exclusion test on day 3, 5 and 7 respectively.

### Cardiomyogenic differentiation

CDCs were divided into three groups i) control, ii) Aza treatment group and iii) Aza+AA treatment group. To induce cardiac differentiation, CDCs were treated with 10 μM Aza for 24 hours in both the treatment groups. After 24 hours, Aza group was maintained in IMDM supplemented with 2% FBS for 14 days, whereas Aza+AA group of cells were maintained in IMDM with 2% FBS and supplemented with 10^-4^M AA every alternative day from 2^nd^ day till the completion of the experiment. During the course of induction, medium was changed regularly and cells were observed for morphological changes. Immunofluorescence analysis and gene expression at transcriptional and translational levels were performed at intervals of 7 and 14 days to measure the extent of cardiac lineage commitment of CDCs [17, 28, 29].

### Growth curve analysis of CDCs

CDCs were seeded in 12 well plates at the density of 5x10^3^ cells/well. After 24 hours, CDCs were divided into control, Aza and Aza+AA groups and treatment was given as mentioned above for the period of 14 days. The growth rate of treated and control CDCs was determined by counting cells manually using trypan blue dye exclusion test on day 1, day 4, day 7, day 11 and day 15 respectively. The growth curves were constructed according to mean values and the graph was plotted with respect to cell number against time of culture (in days).

### Immunofluorescence analysis

Immunofluorescence analysis of control, Aza and Aza+AA treatment groups was performed as described above to check the difference in the expression levels of the following primary antibodies: CD 105, Nkx 2.5, GATA 4 and α-Sarcomeric actinin. To quantify the level of expression among control, Aza and Aza+AA treated CDCs, corrected total cell fluorescence (CTCF) of the cells was calculated using Image J software [26, 30]. Formula for CTCF was as follows: CTCF/μm^2^ = [Integrated density–(area of selected cell x mean fluorescence of background readings)] / Area of selected cell.

### Real time PCR analysis

Total RNA was extracted from control, Aza and Aza+AA treated cells using TRIzol reagent. 1μg of RNA was utilized to synthesize cDNA using Thermofisher Revertaid first strand cDNA synthesis kit according to manufacturer’s instructions. Real time PCR was performed using KAPA SYBR FAST qPCR Master Mix Kit from the synthesized cDNA to analyze the expression of cardiac markers, CD 105, Nkx 2.5 and GATA 4. To know the involvement of Wnt signaling pathway in Aza and Aza+AA mediated differentiation, Wnt direct downstream markers, β-catenin and Cyclin D1 were also analyzed. Primers used for the study are listed in Table 1. The reaction was performed on a StepOne Plus™ Real Time PCR system (Applied Biosystems) and the obtained C_T_ values were normalized to internal control, β-actin and the fold change in gene expression was calculated using the 2^–ΔΔCt^ method.

**Table 1:** Primer sequences used for quantitative real time PCR.

S. No.	Gene	Sense (5’-3’)	Antisense (5’-3’)
1	CD 105	TCAGGCTTACCACCACTTCG	TCACCTTCTGGCCCCAAATC
2	Nkx 2.5	CTTCGTGAACTTTGGCGTCG	TGTGGGTGTGAAATCCGAGG
3	GATA 4	AGCACCCCCTTCCCTCTT	ACCCAGCACCCTTATCCCTA
4	β-catenin	GACCACAAGCAGAGTGCTGA	ACTCGGGTCTGTCAGGTGAG
5	Cyclin D1	TTCTTGTCTCTGGAGCCCCT	ACAACTTCTCGGCAGTCAGG
6	β-Actin	CCATGTACCCAGGCATTGCT	AACGCAGCTCAGTAACAGTCC

### Western blot analysis

Total protein of cells were extracted from control, and the two treated groups of CDCs using RIPA buffer containing 1X protease and phosphatase inhibitor cocktails. Protein concentrations were determined by Bradford assay. 5–40 μg of proteins was separated using 10–12% sodium dodecyl sulfate polyacrylamide gel electrophoresis (SDS-PAGE) and transferred onto Polyvinylidene difluoride (PVDF) membranes using a trans blot apparatus (Bio-Rad, CA, USA). The membranes were blocked with 5% skim milk/bovine serum albumin (BSA) for 1 hour at room temperature and were probed with primary antibodies, GATA 4, α-Sarcomeric actinin, non-phospho β-catenin (active), phospho β-catenin and Cyclin D1 overnight at 4°C. The membranes were washed and incubated with horseradish peroxidase (HRP)-conjugated secondary antibody diluted to 1:8000 in blocking buffer for 1 hour at room temperature. Bands were detected by enhanced chemiluminescence reagent. Band intensities were quantified by Image lab of Image studio Lite software version 5.2 from Licor and normalized to internal control β-actin levels.

### Data analysis

All experiments were performed at least three times and data were expressed as mean ± SEM. Significance between groups was analyzed by One-way/Two-way analysis of variance (ANOVA) or two tailed Student’s *t*-test using GraphPad Prism (version5.0, GraphPad Software, SanDiego, USA). *p*≤0.05 was considered statistically significant in all the cases.

## Results

### Culturing and phenotypic characterization of CDCs

**Fig 1:**
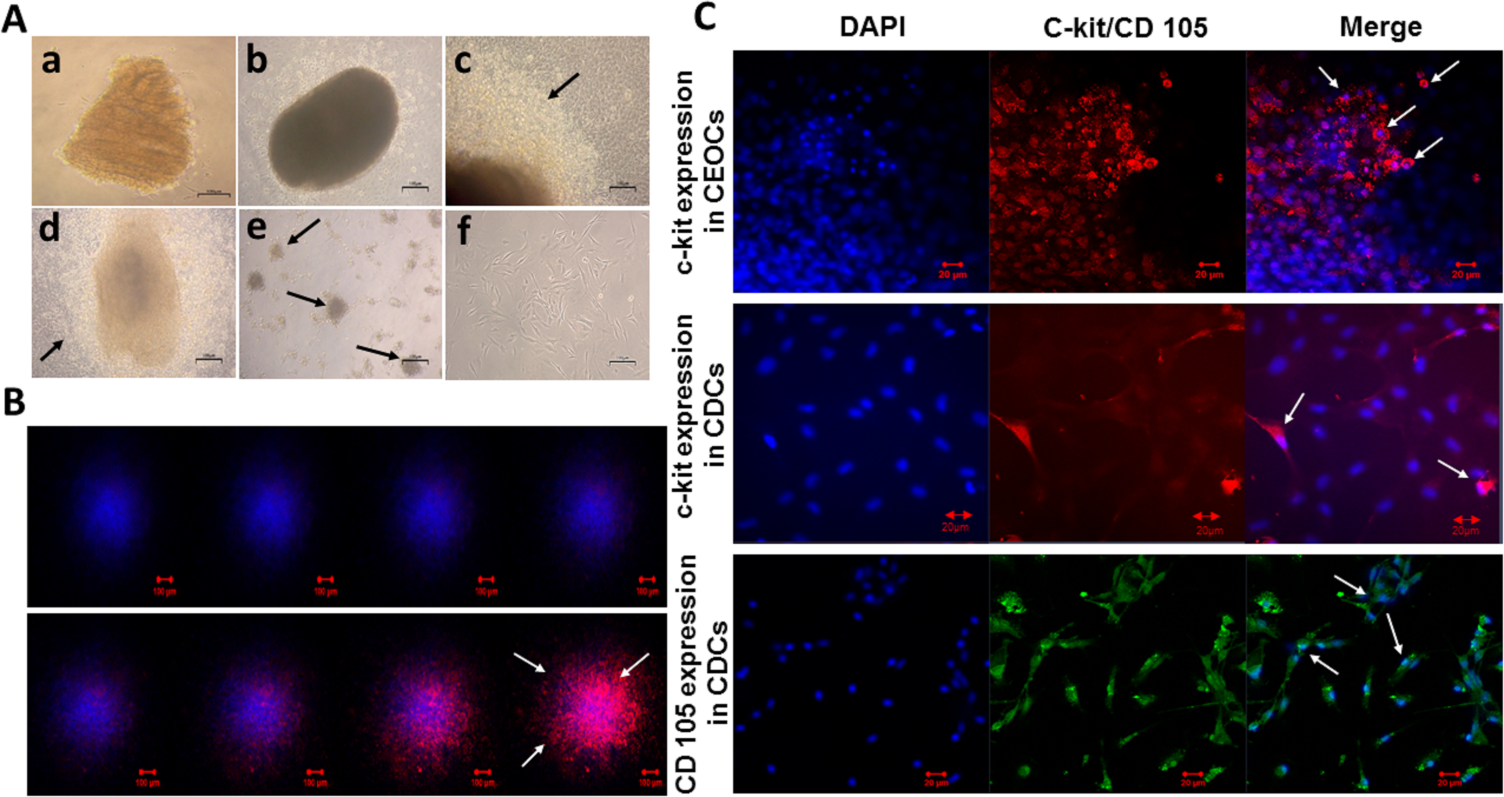
Culture and characterization of CEOCs and CDCs. **(A)** Phase contrast microscopic images representing culture and derivation of cardiosphere derived cells. **a** On day 2 of culture, fibroblast like cells started shedding from the edge of cardiac explants. **b** On day 7 of culture, phase bright round cells were found on top of fibroblast like monolayer around the explants. **c** Robust production of round cells from the explants on day 10 of the culture. **d** Round cells found to be confluent around the explant on 14 days post culture. **e** Formation of Cardiospheres derived from CEOCs. **f** Monolayer culture of CDCs. Arrows indicate the mentioned cells in respective panels. **(B)** Represents immunofluorescence analysis of CEOCs surrounding the explant. Z stacks were acquired for 8 images at 76 μm optical section thickness. CPCs were present in the outermost layer of the explant and were stained positively for c-kit marker (red). While the other cells in the explant were stained with DAPI alone. **(C)** Representative images of CEOCs for c-kit marker and CDCs for c-kit and CD 105 markers. CEOCs showed prominent expression of c-kit whereas CDCs for CD 105 marker. Scale bar = 20μm. Arrows indicate cells positive for the respective marker.

CDCs were obtained by culturing heart biopsies as explants on fibronectin coated culture dishes. A successful outgrowth of fibroblast like cells from cardiac explants was observed after 3–4 days and cell outgrowth was robust with phase bright round cells surrounding the explant by 9–14 days (Fig 1A, a-d). CEOCs were further propagated as cardiospheres to generate CDCs (Fig 1A, e,f). CDCs attained a typical CPC phenotype i.e., spindle shaped morphology.

c-kit, the most widely used marker to identify CPCs was used to analyze CEOCs by immunofluorescence analysis. Z-stack method performed in 8 frames, each with 76 μm thickness displayed c-kit expression only on the surface of the explant. This indicated that the cells shed from the explant were progenitor in nature and freshly shed during culture period (Fig 1B).

Further, CDCs were analyzed for the markers, c-kit and CD 105. CDCs were labelled prominently with CD 105 marker, where as very little expression of c-kit was observed (Fig 1C). Apart from CD 105, CDCs displayed diminutive expression of Nkx 2.5, GATA 4 and α-Sarcomeric actinin proving that they were directed to cardiac lineage.

### Optimization of Aza and AA concentration and their effect on proliferation rate of CDCs

Prior to inducing differentiation of CDCs, optimum concentration and duration of Aza treatment was determined by performing LDH cytotoxicity assay and Alamar blue proliferation assay.

**Fig 2:**
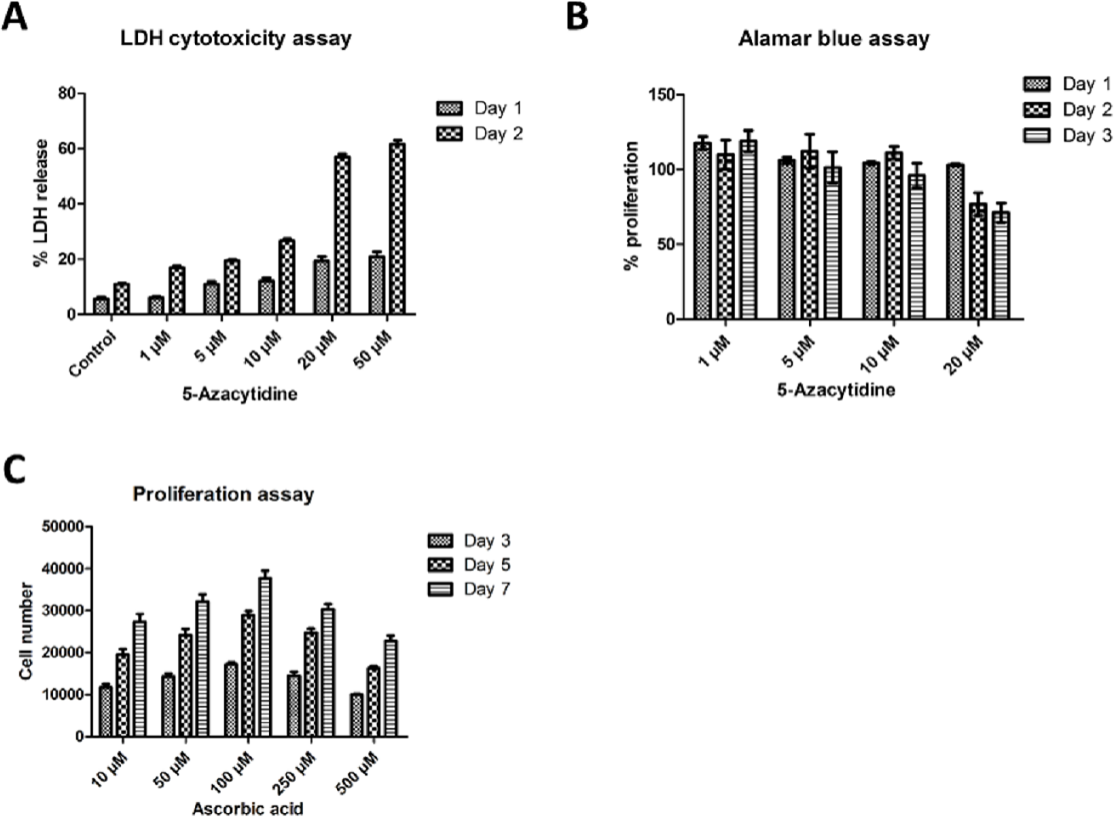
Aza and AA concentration determination. **(A)** Aza concentration determination by LDH cytotoxicity assay. CDCs were treated at five concentrations, (1 μM, 5 μM, 10 μM, 20 μM and 50 μM) of Aza. Significant toxicity of Aza was observed at 20 μM and 50 μM on both day 1 and day 2. Data is presented as Mean ± SEM and the statistical significance (*p* ≤ 0.001) is calculated using Two-way Anova. **(B)** Aza effect on cell proliferation of CDCs determined by Alamar Blue assay. Aza at the concentrations of 1, 5 and 10 μM concentrations did not show distinct difference in proliferation (*p* = ns). Data is presented as Mean ± SEM using Two-way Anova. **(C)** AA concentration determination by proliferation assay. CDCs were treated with 10, 50, 100, 250 and 500 μM concentrations of AA and proliferation rate were assessed using hemocytometer. CDCs displayed increased trend in proliferation from 3 to 7 days at the concentrations, 10, 50 and 100 μM respectively. Data is presented as Mean ± SEM and the statistical significance is calculated using Two-way Anova.

CDCs were treated with 1, 5, 10, 20 and 50 μM concentrations of Aza followed by cytotoxicity assessment after day 1 and 2 of treatment. Obtained results demonstrated that LDH release was minimal at the concentrations of 1, 5 and 10 μM (~10%) on day 1 and therefore were not considered as cytotoxic, while Aza was cytotoxic to cells at the concentrations of 20 and 50 μM (Fig 2A) at both the time points. Further it was observed that there was significant increase in cytotoxicity from day 1 to day 2 at all the concentration ranges (*p*<0.001) with ≥ 50% LDH release to the surrounding medium.

Following LDH assay, effect of Aza on proliferation of CDCs was analyzed by Alamar blue assay at the concentrations of 1, 5 and 10 μM for three consecutive days. As LDH assay displayed significant toxicity at 20 μM and 50 μM, these were omitted for this assay. There was no significant difference in proliferation rate among 1, 5 and 10 μM concentrations (Fig 2B). Hence, the optimal concentration of Aza to induce differentiation of CDCs was determined as 10 μM for one day.

The effect of AA on CDCs proliferation rate was measured manually using haemocytometer on day 3, 5 and 7 of AA treatment. CDCs showed gradual increase in proliferation rate with increase in concentration till 100 μM followed by reversion in proliferative rate in 250 and 500 μM concentrations (Fig 2C).

### Morphological and growth rate analysis of CDCs during differentiation induction

**Fig 3:**
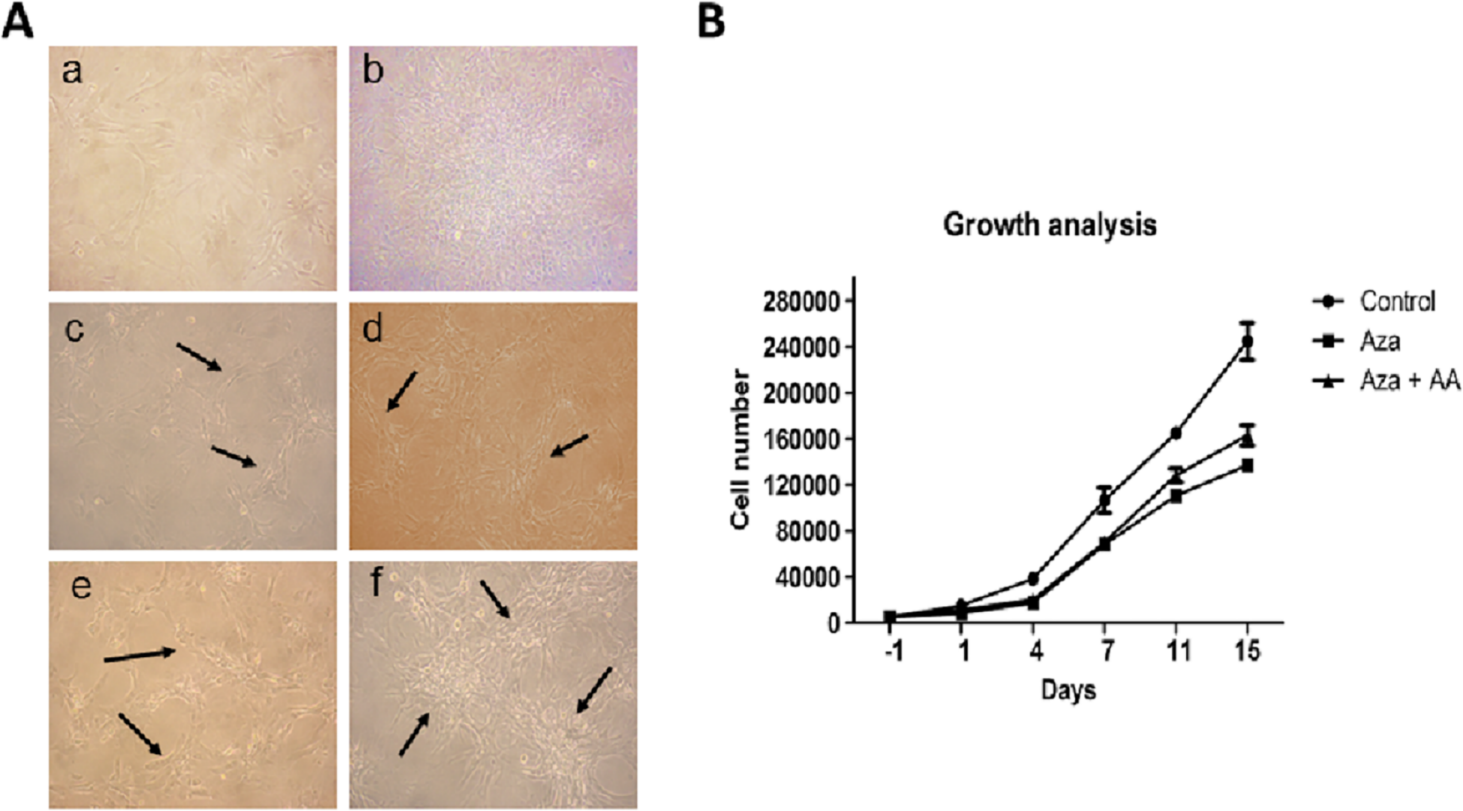
Morphological and growth curve analysis of CDCs during differentiation period. (**A)** Phase contrast images of control, Aza and Aza+AA treated CDCs. **a** and **b** represents control CDCs on day 1 and day 14 of the culture. **c** and **d** represents Aza treated CDCs on day 7 and day 14. Cells became elongated stick like appearance with interconnections between the cells. **e** and **f** represent Aza+AA treated cells with prominent elongated cells. Distinct cell clusters were evident on day 14 of the treatment. (**B)** Growth curve analysis of control and induced CDCs. Control CDCs showed linear progression in proliferation. Proliferative ability of Aza treated cells was less when compared to Aza+AA treated group. Proliferation rate of Aza treated CDCs were significantly less when compared to control CDCs (*p<0.05).

Phase contrast images represented that CDCs maintained spindle shape throughout the course in control cells (Fig 3A, a & b). In Aza treatment groups, cells became elongated with stick like appearance (Fig 3A, c) and cell interconnections between the cells were observed by 14 days of treatment period (Fig 3A, d). On 7^th^ day of induction, CDCs treated with Aza+AA (3A, e) showed notable interconnections between the cells with formation of distinct cell clusters (3A, f) on 14^th^ day of the culture.

Growth curve analysis revealed that, in comparision to control, there was a distinct decrease in proliferation ability in both the treatment groups, mainly in Aza group (*p* = 0.0245). During the differentiation period, while control CDCs maintained logarithmic proliferation, both Aza and Aza+AA groups showed decreased proliferative rate with lag phase till 5–7 days of culture (Fig 3B). After 7 days, Aza+AA group showed higher proliferation rate than Aza group (*p* = 0.1123).

### AA significantly enhanced Aza induced differentiation of CDCs to cardiomyogenic lineage

**Fig 4:**
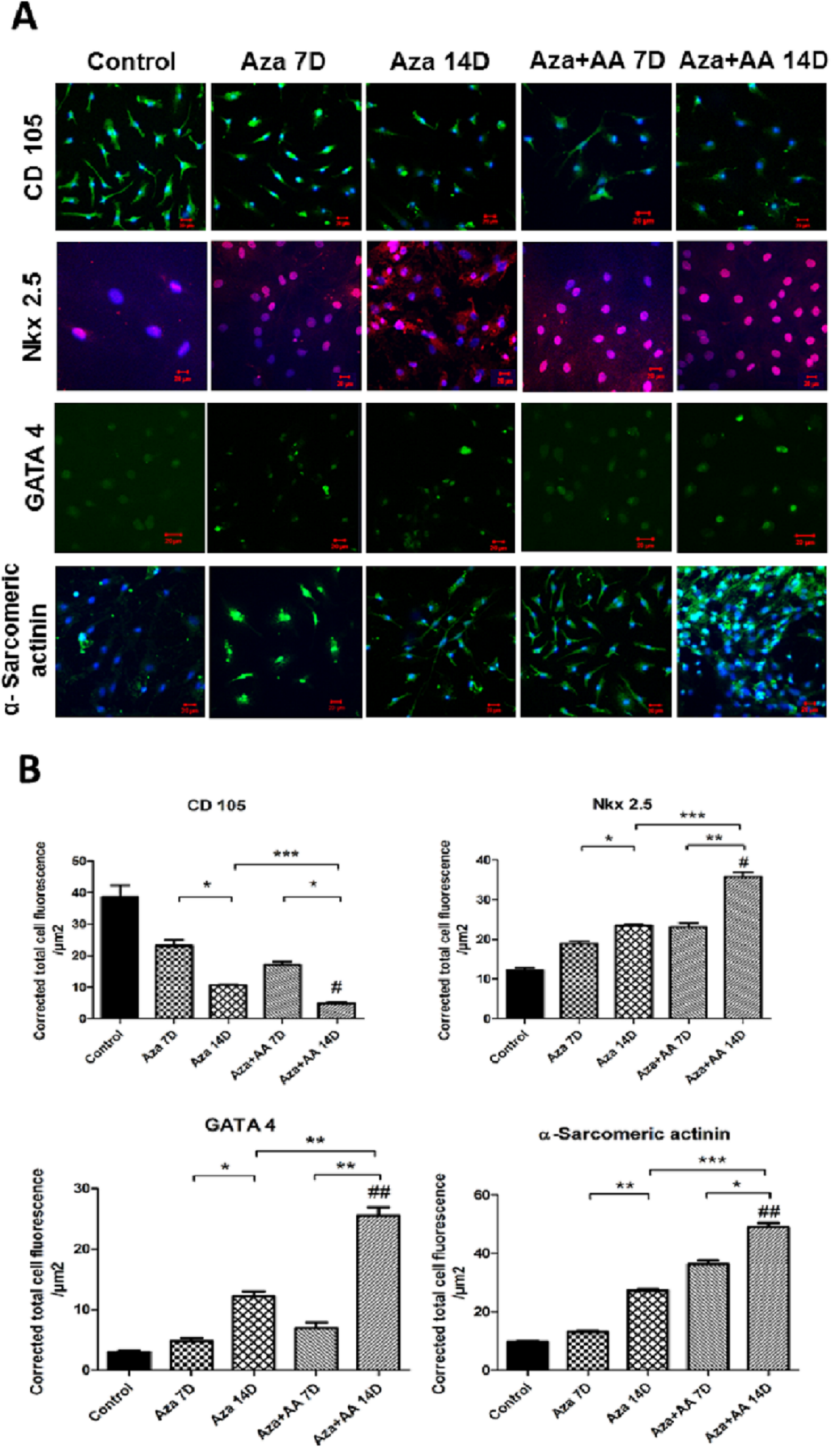
Immunofluorescence analysis of control, Aza and Aza+AA treated CDCs. (**A)** Immunofluorescence analysis of control and treated CDCs for markers–CD 105, Nkx 2.5, GATA 4 and α-Sarcomeric actinin respectively. Prominent decrease in expression of CD 105 was found in Aza+AA group (*p*≤0.05). Whereas cardiac lineage markers, Nkx 2.5, GATA 4 and α-Sarcomeric actinin showed high expression in both the treatment groups with major enhancement in Aza+AA group. (**B)** Quantitative evaluation of CDCs differentiation using corrected total cell fluorescence measurement. # represents overall significance with respect to control as analyzed by One way Anova with Kruskal Wallis test. * represents significant values between groups using student’s *t*-test analysis. #*p*< 0.05, *##p* <0.01. *p < 0.05, ** *p* < 0.01, *** *p* < 0.001 respectively.

CDCs were analyzed for the markers, CD 105, Nkx 2.5, GATA 4 and α-Sarcomeric actinin to investigate if Aza and Aza+AA have driven them to cardiac lineage (Fig 4A) CTCF measure displayed that CD 105 marker was highly expressed in control group when compared to both the treatment groups by the end of 14 days (*p* = 0.027). Aza+AA group showed a significant low expression (*p* = 0.0003) compared to Aza group. Cardiac transcription factors, Nkx 2.5 and GATA 4 showed augmented expression in Aza+AA group (*p* = 0.0118 and *p* = 0.009 respectively) as compared with control. Both the markers showed marked expression in Aza+AA group (*p* = 0.0006 and *p* = 0.0012) than Aza group by 14 days of the treatment. α-Sarcomeric actinin expression was highly significant (*p* = 0.0091) in Aza+AA group at the end of the treatment not only when compared to control but also when compared to Aza group (*p* = 0.0001) (Fig 4B). Obtained results clearly displayed a phase transition from a progenitor stage to a stable cardiomyogenic lineage.

### Aza+AA efficiently activated mRNA expression of cardiomyogenic markers in CDCs

**Fig 5:**
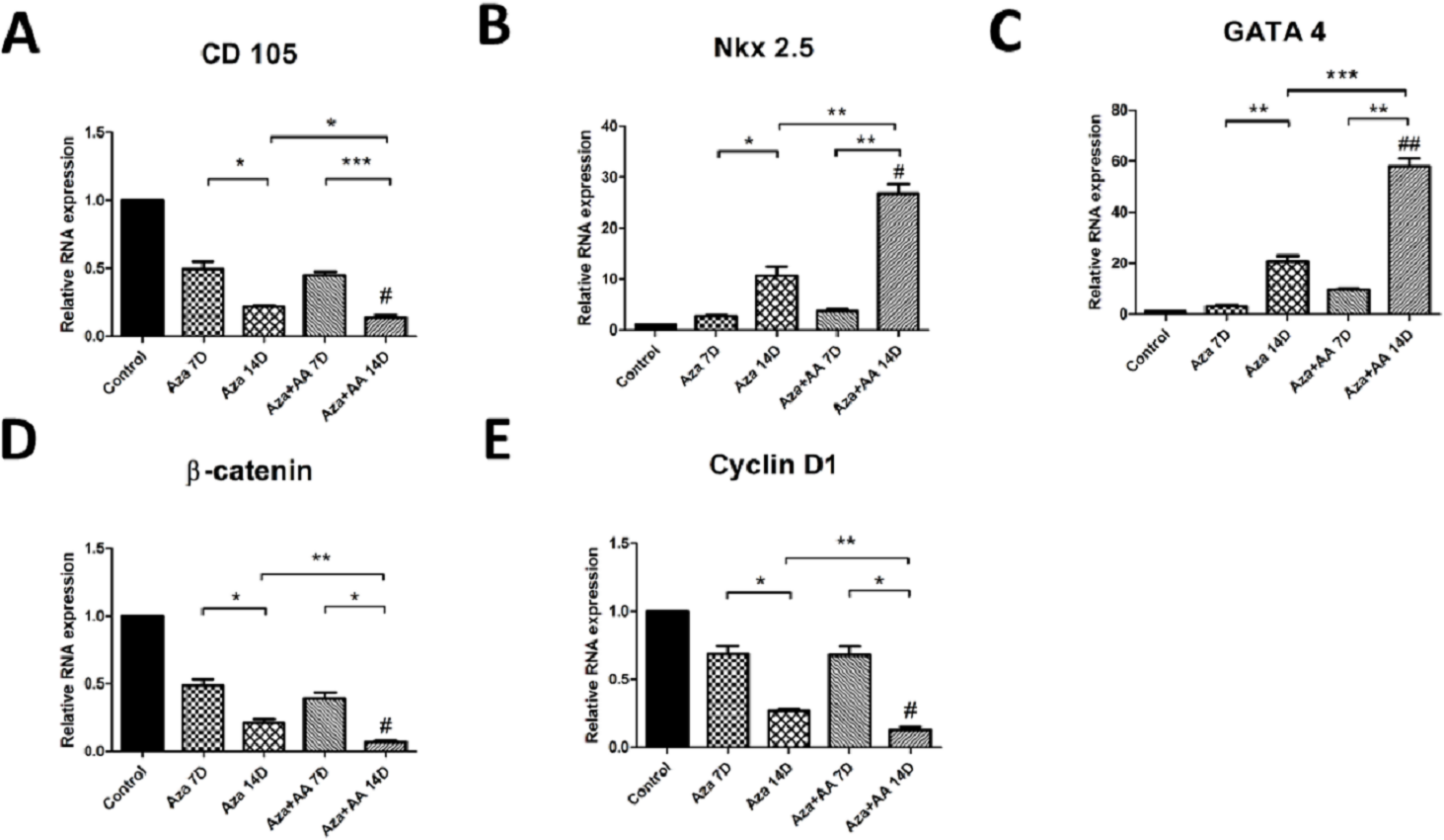
Real time PCR analysis of control, Aza and Aza+AA treated CDCs. (**A)** CD 105 showed distinct down regulation in Aza+AA treated CDCs when compared to control on 14^th^ day (#*p* < 0.0241). (**B**-**C)** Nkx 2.5 and GATA 4 expression were relatively upregulated in Aza+AA group when compared to control on 14^th^ day (#*p* < 0.0156 and *p* < 0.0087 respectively). (**D-E)** β-catenin and Cyclin D1expression were distinctly down regulated in Aza+AA group when compared to control with *p* values, 0.0107 and 0.0116 respectively. # represents overall significance with respect to control as analyzed by One way Anova with Kruskal Wallis test. * represents significant values from student’s *t*-test analysis. #, *p* < 0.05 Vs control, *p < 0.05, **p < 0.01, ***p < 0.001.

To check if Aza and Aza+AA has directed CDCs to cardiac lineage, the expression levels of CD 105, Nkx 2.5 and GATA 4 were evaluated by real time PCR. There was a significant down regulation of CD 105 expression in both the treated groups compared to control (*p*<0.05) (Fig 5A). Substantial decrease in expression of CD 105 was observed from 7 to 14 days on both the treated groups especially in Aza+AA (*p* = 0.0009). Early cardiac transcription factor, Nkx 2.5 showed distinctly relative upregulation in Aza+AA group (*p* = 0.0048) from 7 to 14 days than Aza group (*p* = 0.0462), with significant difference between Aza and Aza+AA groups by 14^th^ day of treatment (*p* = 0.0034) (Fig 5B). Another cardiac transcription factor, GATA 4 displayed significant upregulation in expression by 14 days in Aza+AA group (*p* = 0.0006) with a fold increase of 60 whereas the Aza group showed a 20-fold increase against control (Fig 5C). To check if Aza and Aza+AA influence Wnt signaling pathway, β-catenin and Cyclin D1 expressions were also evaluated. β-catenin expression was prominently downregulated in Aza+AA group (*p* = 0.024) when compared to Aza (*p* = 0.0377) from 7 to 14 days. Significant difference (*p*<0.01) in β-catenin expression was observed between treatment groups on 14^th^ day (Fig 5D). Similarly, Cyclin D1 expression was significantly down regulated in Aza+AA (*p* = 0.0185) as compared with Aza (*p* = 0.0206), with a significant difference in expression levels between the treatment groups (*p*<0.01) (Fig 5E). The results have shown that Aza along with AA upregulated cardiac genes by negatively influencing Wnt pathway target genes and aid in directing CDCs to cardiomyogenic lineage.

### Protein expression analysis revealed that Aza+AA prominently activated cardiac specific markers in CDCs by inhibiting the Wnt signaling pathway

**Fig 6:**
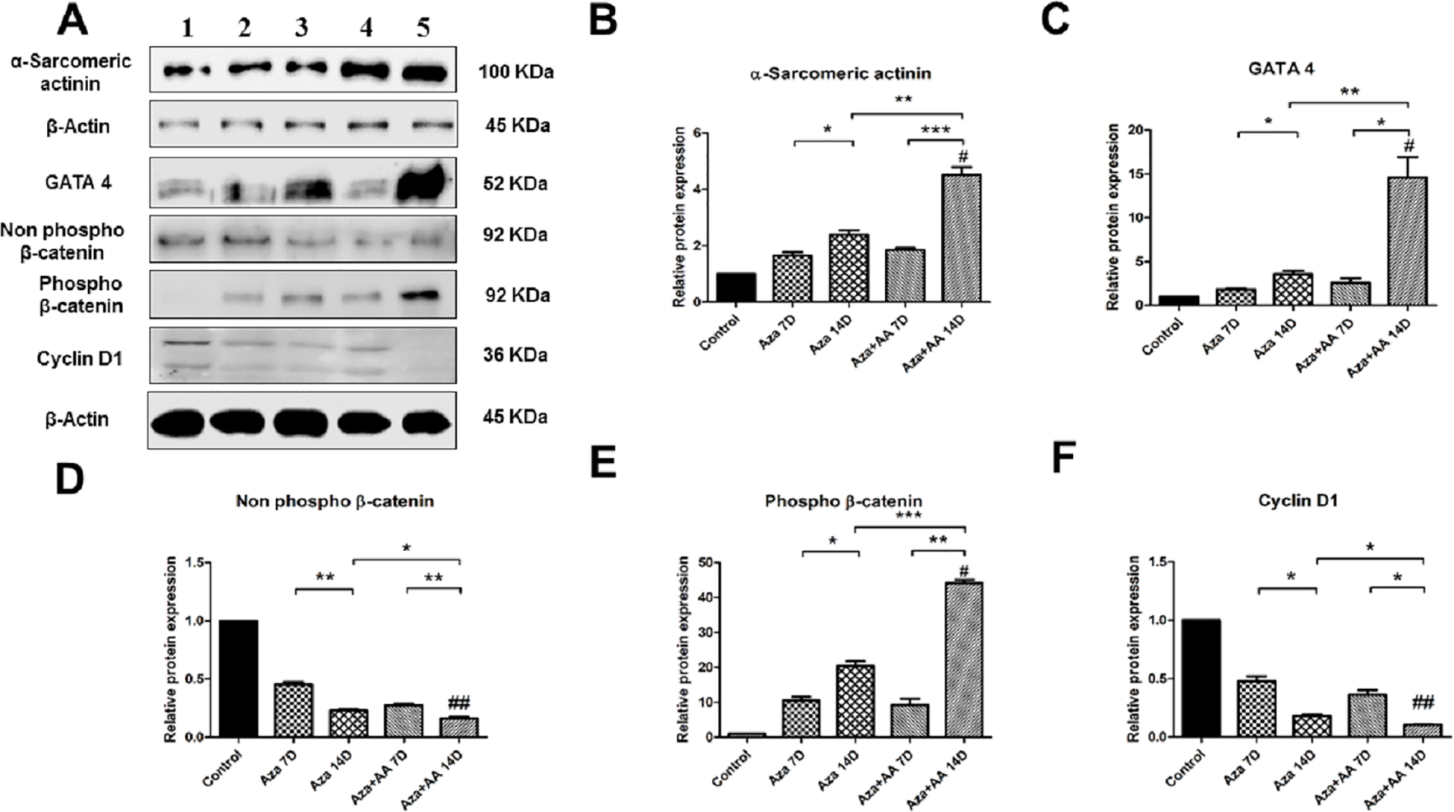
Protein expression in control, Aza and Aza+AA treated CDCs. (**A)** Representation of protein expression by Western blot analysis - α-Sarcomeric actinin, GATA 4, non-phospho β-catenin, phospho β-catenin and Cyclin D1. Lane 1 = control, Lane 2 = Aza 7 days, Lane 3 = Aza 14 days, Lane 4 = Aza+AA 7 days, Lane 5 = Aza+AA 14 days. (**B-E)** Graphical representation of the respective proteins expression in comparison to control. α-Sarcomeric actinin showed significant upregulation in Aza+AA group (*p* ≤ 0.001). GATA 4 displayed marked upregulation in expression on 14^th^ day of Aza+AA group (*p* = 0.0242). Wnt proteins, non-phospho β-catenin and Cyclin D1 showed significant down regulation by 14 days in both the treatment groups, especially in Aza+AA group (*p* ≤ 0.05) while phospho β-catenin showed significant up regulation on 14^th^ day in Aza+AA treatment group (*p* = 0.0001) when compared to Aza. # represents overall significance with respect to control. * represents significance of student’s t-test analysis. #*p* < 0.05 Vs control, *p ≤ 0.05, **p ≤ 0.001, ***p ≤ 0.0001.

In western blot, one cardiac transcriptional factor (GATA 4) and one structural protein (α-Sarcomeric actinin) were chosen for analysis. Also, β-catenin was analyzed in its two forms, non-phospho β-catenin (active) and phospho β-catenin were analyzed in order to know the distinct role of β-catenin in differentiation. In line with RT-PCR results, Western blot confirmed increased expression of GATA 4 and α-Sarcomeric actinin and relative down regulation of Wnt signaling pathway related proteins, non-phospho β-catenin and Cyclin D1 with upregulation of phospho β-catenin (Fig 6A).

Cardiac structural protein, α-Sarcomeric actinin showed timely progression in its expression from 7 to 14 days in both the treatment groups (Aza, *p* = 0.0198 and Aza+AA, *p* = 0.0007 respectively) when compared to control with overall significance of *p*≤0.01. A significant difference in expression was observed by 14^th^ day between Aza and Aza+AA groups (*p* = 0.0024) (Fig 6B). GATA 4 has showed prominent expression in Aza+AA group from 7 to 14 days of treatment (*p* = 0.0242) as compared to Aza group (*p* = 0.0319) with overall significance of *p =* 0.0123 when compared to control (Fig 6C). Non-phospho β-catenin showed downregulation in both Aza and Aza+AA group from 7 to 14 days (*p* = 0.002 and 0.008 respectively) with significant difference between the groups by 14^th^ day (*p* = 0.0205) (Fig 6D). Cyclin D1 showed marked decrease in expression on 14^th^ day of induction in Aza+AA group (*p* = 0.0254) as compared to Aza group (*p* = 0.0339). The difference in Cyclin D1 expression between the groups was found significant (*p* = 0.0113) (Fig 6F). Interestingly, the protein expression level of phospho β-catenin was very much significant in Aza+AA group on 14^th^ day of induction (*p*<0.001) when compared to Aza group. Aza+AA group showed significant increase (*p* = 0.002) in expression of phospho β-catenin from 7 to 14 days when compared to Aza group alone (*p* = 0.0105) (Fig 6E).

### Cardiomyogenic potential of CDCs

Aza+AA induction resulted in development of spontaneous generation of beating in areas with cell aggregates. The beating of cells was observed on the 10^th^ day of culture and was observed till the time of treatment termination. **S1 Video** (supporting information) demonstrated the beating at a lower pace (1 beat/4 seconds) on day 10 of culture. **S2 Video** (Supporting information) demonstrated the persistence of beating on day 14 of the culture. Interestingly the beating rate has been increased to 2 beats/sec when compared to day 10. Cells surrounding the beating area also got synchronized and showed rapid contractions as observed on the day of termination. This substantiated the ability of Aza+AA in directing CDCs to cardiomyogenic lineage than Aza alone treatment.

## Discussion

**Fig 7:**
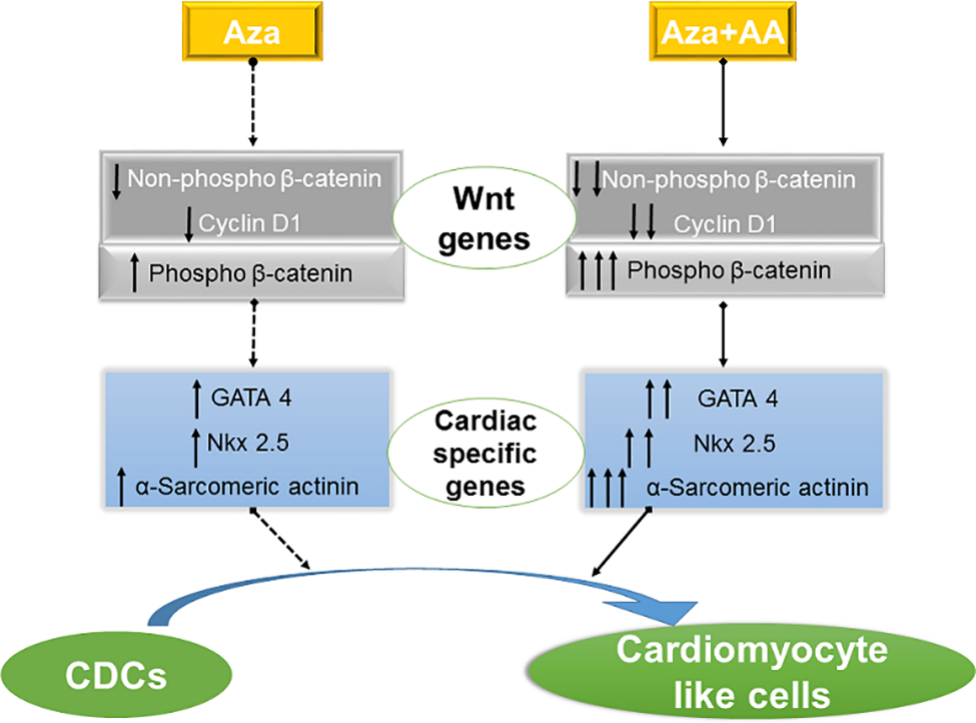
Schematic representation of CDCs differentiation towards cardiomyogenic lineage. Aza+AA efficiently differentiated CDCs to cardiomyocyte like cells with distinct upregulation of cardiac specific genes. Wnt pathway was negatively regulated in this process via phosphorylation of β-catenin.

The conventional treatments for cardiovascular diseases can prevent the progression of the disease but are unable to restore/regenerate the infarcted myocardium. Stem cell therapy represents an alternative approach to replenish the damaged myocardial tissue. CDCs are one of the ideal candidates for myocardial regeneration since they are endogenous with no known adverse immunological reactions. Even though reports on transplantation of CDCs in animal models and clinical trials were encouraging, not much improvement in cardiac function was observed. Issues like low cell survival (<10%), minimal differentiation ability and poor engraftment of CDCs show that therapeutically it is still in nascent stage [9, 13, 31, 32]. These pitfalls associated with CDCs may be swept over by pre-implantation differentiation. Henceforth, we stimulated CDCs with combination of small molecules (Aza alone and in combination with AA) to analyze their fate-conversion towards cardiomyogenic lineage. In the present study, CDCs from rodent hearts were derived, characterized and their differentiation potential were investigated through the downstream regulators of Wnt signaling pathway (Fig 7, schematic representation).

CDCs are progenitor cells of heart derived by explant culture and mainly comprised of subpopulations of cells with markers of cardiac progenitor cells (c-kit+/ CD90−), mesenchymal stem cells (c-kit−/CD 105+,90+) and endothelial cells (c-kit−/CD34+), which show synergistic effect on myocardial regeneration [7]. In this study, we demonstrated via immunofluorescence analysis that CDCs showed distinctly elevated expression for CD 105 but with low levels of c-kit. Our results were consistent with previous studies, that CDCs express primarily CD 105 marker (75–95%) and very low percentage of cells positive for c-kit (1–5%) [10, 11, 33, 34]. Study by Davis et al had proved that freshly shed outgrowth cells from heart explants were of non-cardiomyocyte origin using a bi-transgenic mouse model containing GFP labelled cardiomyocytes [8]. In line with this, our analysis of explant outgrowth by Z-stack method via immunofluorescence analysis showed that CEOCs express c-kit marker only in the freshly shed outgrowth cells validating that no cardiomyocyte contamination was present.

Recently, small molecule mediated differentiation of stem cells has gained much interest in stem cell field. This method has added advantages of being economical, safe and effective when compared to growth factors or cytokines. Moreover, biological activities can be easily controlled with little side effects and help to understand the signaling pathways involved in generating differentiated cells [14]. Aza, a demethylating agent is a potential myogenic inducer and is shown to reactivate silenced myogenic markers expression during differentiation process [35, 36]. It is a chemical analogue of cytosine which acts by inhibiting methyltransferases thereby selectively activating gene expression in a short span of time [37]. Hence we believe that exposure of CDCs to Aza for a period of 24 hours is much more sufficient to reactivate cardiac specific genes in CDCs without exerting any toxicity on cells. AA is known to enhance the proliferation of CPCs by increasing collagen synthesis via MEK-ERK1/2 pathway and induce cardiac differentiation by upregulating the late-stage markers of cardiogenesis [38, 39]. Nevertheless, Aza is known to induce some cytotoxicity apart from its inducing effect, while AA exerts its effect on proliferation with divergence in differentiation ability [19].

We hypothesized that AA may promote the differentiation of CDCs induced by Aza. Before inducing differentiation of CDCs, Aza and AA concentrations were determined to ensure no toxicity during treatment period. AA showed its effect mainly on proliferation, but no significant induction of differentiation rate was observed (data not shown). Growth curve analysis revealed better proliferation rate in Aza+AA than Aza treatment alone. Previously, it was mentioned that AA might show its proliferation enhancing ability after 2–6 days of culture initiation [10]. Our result demonstrated that cells treated along with AA showed enhanced proliferation from 7^th^ day onwards compared to Aza treatment alone.

Results from immunofluorescence, real time PCR and western blot validated that Aza+AA treatment group possessed higher myogenic induction potential and could effectively direct CDCs towards cardiomyogenic lineage than Aza alone. The CDC specific progenitor marker, CD 105 expression was suppressed significantly in treated groups, particularly in Aza+AA. Immunofluorescence analysis specified 20-fold decrease of CD 105 expression in Aza+AA group (*p* = 0.0003) than Aza group with over all significance of *p* ≤ 0.05 compared to control. At the mRNA level also Aza+AA group displayed a 9-fold decrease in expression (*p =* 0.0184) when compared to control. Overall, it was confirmed that CDCs were directed from a progenitor to cardiomyogenic phase.

The homeobox transcription factor, Nkx 2.5 and the Zinc-finger transcription factor, GATA 4 are the two well characterized transcription factors which usually co-express in early cardiogenesis events [40]. Immunofluorescence analysis had shown 15-fold augment in Nkx 2.5 and GATA 4 expression levels in Aza+AA group compared to Aza alone. Whereas, RNA levels demonstrated 40-fold increase in GATA 4 expression compared to Nkx 2.5 which showed a 20-fold change (p≤0.001 between Aza+AA and Aza groups on 14^th^ day) during stimulation along with AA. Takahashi et al has demonstrated that AA significantly induce GATA 4 expression in cardiac differentiation of ESCs. In accordance with this, we propose that AA might have synergistic effect in relative upregulation of GATA 4 in Aza+AA treated CDCs when compared to Aza alone. Western blot experiments had also confirmed a 10-fold enhanced GATA 4 expression in the presence of AA. Also, both immunofluorescence and Western blot confirmed that there was a significant increase in the expression of cardiac structural protein, α-Sarcomeric actinin in Aza+AA group than Aza (*p*≤0.0001). Arminan et al have revealed a direct correlation between the α-Sarcomeric actinin expression and nuclear translocation of Nkx 2.5 and GATA 4 [41].

In this study, along with the ability of differentiating CDCs, we also investigated Aza and AA’s role in modulating Wnt/β-catenin pathway. RNA expression results revealed that Aza+AA greatly suppressed the expression of β-catenin and cyclin D1 levels by 14 days of treatment (*p* ≤ 0.01). Wnt/β-catenin signaling coordinates stem cell maintenance, proliferation and cell-fate decisions both at fetal and adult life. Proteolytic regulation of β-catenin, is the crucial step in modulating Wnt/β-catenin signalling cascade. In the absence of Wnt signals, the destruction complex binds and phosphorylates cytosolic β-catenin at N-terminus resulting in its degradation by the ubiquitin–proteasome system and therefore the level of active, unbound cytosolic β-catenin is maintained at very low levels [42–43]. In western blot, we checked the expressions of phospho β-catenin and nonphospho β-catenin. Phospho β-catenin expression would be detected only if it gets phosphorylated at serine 33, 37 and threonine 41 sites. Phosphorylation of these sites on β-catenin was mediated by GSK-3β leading to less/poor accumulation of non-phospho β-catenin (active form) in the nucleus resulting in the inhibition of the Wnt pathway [44]. From our results, we are able to state that Aza+AA might have caused differentiation by mediating phosphorylation at the sites, serine 33, 37 and threonine 41 on β-catenin leading to its proteolytic degradation.

Wnt signaling pathway maintains stemness and positively regulates proliferation of stem cells and is known to negatively regulate differentiation [45]. Moreover, there have been reports demonstrating that down regulation of β-catenin is necessary for cardiac differentiation and this in turn associated with enhancement of cardiac markers, Tbx5 and GATA 4 expression [23]. These data support the results obtained in this study is that, GATA 4 and β-catenin were negatively related to each other. Study involving anti Nkx 2.5 miRNA and Wnt ligands revealed that over expression of β-catenin repress Nkx 2.5 and also GATA 4 expression in CPCs [46]. Moreover, Nkx 2.5 show negative regulation in Wnt signaling during early cardiomyogenesis [47]. In accordance with this, our results also suggest that Aza and AA induce CDC differentiation by negatively influencing Wnt pathway.

Overall, these outcomes suggest that stimulation with Aza followed by AA supplementation has a synergistic effect in cardiomyogenic induction from CDCs making them an ideal candidate for cardiac regenerative therapeutics. It was also proved that AA enhanced the effect of Aza mediated generation of cardiomyocytes from CDCs. Aza+AA promoted CDCs cardiac lineage commitment by down regulating Wnt signaling pathway. Further *in vivo* experiments are prerequisite to evaluate the role of Wnt/β-catenin pathway in Aza+AA mediating differentiation.

## Conclusion

To conclude, successful induction to cardiomyocytes depends on the type of treatment given to progenitor cells. Our *in vitro* studies suggest that stimulation of CDCs with Aza followed by AA treatment can prominently enhanced proliferation and differentiation of CDCs with down regulation of Wnt pathway by phosphorylating β-catenin. We are the first group to report spontaneous beating of CDCs by Aza and AA mediated differentiation. These findings reveal the possible role of CDCs in cardiac repair and provides us with new perceptions in the area of cardiac regeneration.

## Supporting information

S1 Video Development of spontaneous beating in Aza+AA treated CDCs on 10^th^ day of the differentiation induction. Beating rate was observed as 1 beat per four seconds.
(MP4)

S2 Video Persistence of beating of Aza+AA treated CDCs on 14^th^ day of culture. When compared to 10^th^ day, cells surrounding the initial beating area were also synchronized in beating with enhanced pulse of 2 beats per second.
(MP4)
